# Impact of Surface States and Aluminum Mole Fraction on Surface Potential and 2DEG in AlGaN/GaN HEMTs

**DOI:** 10.1186/s11671-021-03615-x

**Published:** 2021-10-20

**Authors:** Pragyey Kumar Kaushik, Sankalp Kumar Singh, Ankur Gupta, Ananjan Basu, Edward Yi Chang

**Affiliations:** 1 Center for Applied Research in Electronics (CARE), IIT Delhi, New Delhi, India; 2 International College of Semiconductor Technology, National Chiao Tung University, Hsinchu, 1001 Taiwan

**Keywords:** Gallium nitride (GaN), High-electron-mobility transistor (HEMT), Two-dimensional electron gas (2-DEG), Surface traps

## Abstract

The presence of surface traps is an important phenomenon in AlGaN/GaN HEMT. The electrical and physical properties of these surface traps have been analyzed through the study of 2DEG electron concentration along with the variation of aluminum percentage in the barrier layer of HEMT. This analysis shows that from deep to shallow donors, the percentage change in electron density in 2DEG gets saturated (near 8%) with change in aluminum concentration. The depth of the quantum potential well below the Fermi level is also analyzed and is found to get saturated (near 2%) with aluminum percentage when surface donor states energy changes to deep from shallow. The physics behind this collective effect is also analyzed through band diagram too. The effect of surface donor traps on the surface potential also has been discussed in detail. These surface states are modeled as donor states. Deep donor (*E*_C_ *− E*_D_ = 1.4 eV) to shallow donor (*E*_C_ − *E*_D_ = 0.2 eV) surface traps are thoroughly studied for the donor concentration of 10^11^ to 10^16^ cm^−2^. This study involves an aluminum concentration variation from 5 to 50%. This paper for the first time presents the comprehensive TCAD study of surface donor and analysis of electron concentration in the channel and 2DEG formation at AlGaN–GaN interface.

## Introduction

High-frequency and high-power applications are the two main traits of the GaN material which have been studied in the last three decades [1, 2]. One of the main advantages of AlGaN/GaN structure is the formation of 2DEG in the triangular potential well at AlGaN–GaN interface even without intentional doping in the barrier layer [3, 4]. It is well proven that spontaneous and piezoelectric polarization exists in the AlGaN layer of AlGaN/GaN structure [3]. This polarization results into two opposite sheets of charges at the bottom and top of the AlGaN barrier layer. These polarization sheet charges alone are not sufficient to form a triangular potential well at the AlGaN–GaN interface. In order to address this, Ibbetson et al*.* [5] suggested that there should be a positive sheet of charges which has to exist at the surface of AlGaN layer. These positive charges pop up due to the ionization of the surface donor states (1.42 eV from conduction band with 1.35 × 10^13^ cm^−2^) at the surface [6].

Vetury et al*.* [7] investigated the effect of these surface states using potential probes as the floating gate. The effect of surface states on DC and RF performance of AlGaN/GaN HEMT has been studied [8, 9]. Nanometer-scale Schottky gate behavior discusses the virtual gate formation in the un-gated region due to surface donor states [10]. The fixed surface donor states are used to analyze the self-heating effect in HEMT [11]. Longobardi et al*.* [12] performed the first TCAD simulation to study the effect of surface donor states on the DC characteristics of AlGaN/GaN MISFETs. To activate these surface donor states in the TCAD simulation, Bakeroot and others introduced a different model [13, 14]. Drain/Source resistances are also dependent on gate bias due to the formation of the virtual gate in the un-gated region of the AlGaN surface. Pradeep et al*.* [15] have developed the mobility and resistance extraction procedure based on linear region DC characteristics of AlGaN/GaN HEMT. Meneghesso et al*.* [16] discussed the surface state as a trap which captures the highly dense hole layer on the surface of AlGaN to compensate electrons in 2DEG. The surface donor traps available at the top of the AlGaN layer alter the electrical behavior of the device when these traps are occupied by electrons with negative gate bias [17]. The relation between surface donor traps and 2DEG electrons has also been discussed through TCAD simulation by adopting time-dependent transport phenomena [18]. Though different characterization techniques have been explored, Tapajna et al. [19] used threshold-transient method to investigate the interface acceptor traps, but surface donor traps characterization is still unexplored. An extensive computational modelling approach for the surface trap as a donor has also been discussed [20]. Gucmann et al*.* [21] discussed that if the density of surface donor is greater than the polarization charge concentration, then electron get transferred to the AlGaN–GaN interface to originate the 2DEG into the channel.

The above-discussed literature has reported so many relevant aspects of AlGaN/GaN heterostructure but does not account for the combined effect of the surface donor (Concentration + Energy) and contribution of aluminum concentration in the two-dimensional electron concentration. It is evident that aluminum percentage is primarily responsible for polarization charge in AlGaN barrier layer [3].

To provide an appropriate physical understanding of such an effect, we have covered following investigation in the present work (i) the effect on two-dimensional electron concentration with surface donor trap changes from deep to shallow along with aluminum percentage changes in AlGaN barrier layer, (ii) the effect of surface trap and aluminum percentage on surface potential and (iii) the influence of surface donor trap and aluminum percentage on the triangular potential well at AlGaN–GaN interface.

## Method Section and Simulation Setup

**Fig.1:**
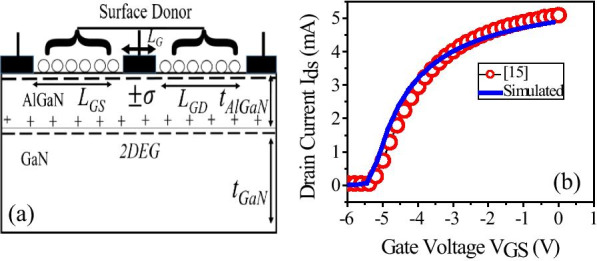
**a** Schematic of 2D simulated structure with Al mole fraction of 28%. **b** Calibration of the simulation setup by reproducing the experimental results reported [15]

2-D device simulations were carried out using Synopsys’s Sentaurus TCAD version L-2016.12 [22]. We calibrated the TCAD simulation setup by reproducing the experimental result of AlGaN/GaN HEMT heterostructure [15], as shown in Fig. 1b.

The calibrated device has 30 nm AlGaN barrier layer on the top of the 2 μm GaN buffer layer. Schottky gate length (*L*_G_) of 1 μm is placed symmetrically on the top of the AlGaN barrier layer. Un-gated region from gate to drain/source (*L*_GD_/*L*_GS_) has a dimension of 2.5 μm each and width of the device is 150 μm.

The simulation platform tackles three conditions (Poisson condition alongside electron and hole continuity condition) which oversees the semiconductor behavior.

The self-consistent drift and diffusion (DD) transport model is also included. Lombardi mobility and Philip unified mobility model were invoked to facilitate mobility degradation due to the electric field and doping. In addition, Auger and SRH (Shockley–Read–Hall) recombination model was used along with Fermi–Dirac statistics. Slotboom model is activated to encounter the bandgap narrowing of heavily doped drain and source extended area. Since this structure has two layers and we are changing the aluminum percentage in the AlGaN barrier layer, the polarization charge is introduced according to the equation of [3]:

1 $$\left| {\sigma (x)} \right| = \left| {2\frac{a(0) - a(x)}{{a(x)}}\left\{ {e_{31} (x) - e_{33} \frac{{C_{13} (x)}}{{C_{33} (x)}}} \right\} + P_{{{\text{SP}}}} (x) - P_{{{\text{SP}}}} (0)} \right|$$

where *P*_SP_ is spontaneous polarization, *e*_33_ and *e*_31_ are piezoelectric coefficients, *C*_33_ and *C*_31_ are elastic constants, a is the lattice constant and *x* is a mole percentage of aluminum.

**Fig. 2:**
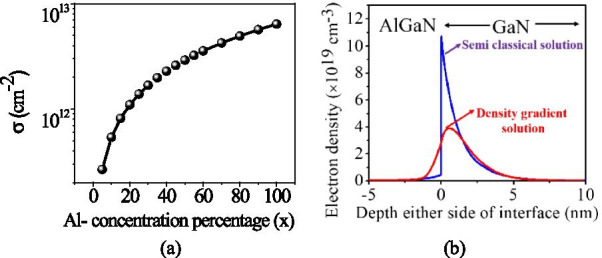
**a** Variation of polarization sheet charge with respect to the aluminum concentration in the AlGaN barrier layer [3]. **b** Effect of quantum captivity on 2DEG electron density

The variation of polarization charge ± *σ*_AlGaN_ (*x*) with aluminum percentage is depicted in Fig. 2a [3]. Once the polarization charge is calculated, the Poisson equation can be solved. At AlGaN–GaN interface conduction band changes abruptly and forms a narrow (1–4 nm) triangular potential quantum well where electrons accumulate. As this quantum potential well very narrow, the reduced density of the states becomes dominant. Schrodinger quantum equation accounts for the quantum potential well but is difficult to solve for a larger HEMT device. To capture the quantum potential well behavior, we invoked eQuantumpotential model in the Sentaurus TCAD which activates the density-gradient quantum correction model [23] and gives a close matched result with the Schrodinger quantum equation for larger HEMT devices (power HEMT devices). The density-gradient quantum model reduces the peak value of electron density in 2DEG, and the peak value also shifts away from the AlGaN–GaN interface. Hence, this reduces the interface scattering mechanism and improves the mobility in the channel see Fig. 2b [20]. Density-gradient quantum model introduces an extra term Λ into normal density formula like:

2 $$n = N_{{\text{C}}} F_{1/2} \left( {(E_{{\text{F}}} - E_{{\text{C}}} - \Lambda )/kT} \right)$$

where *N*_C_ is the effective density of states, *F*1*/*2 is Fermi integral of order 1*/*2, *E*_F_ is the quasi-Fermi energy for electrons, *E*_C_ is the conduction band edge and *kT* represents thermal energy of electrons. Λ is calculated by:

3 $$\Lambda = - \left( {\left( {{{\gamma \hbar^{2} } \mathord{\left/ {\vphantom {{\gamma \hbar^{2} } {6m_{n} }}} \right. \kern-\nulldelimiterspace} {6m_{n} }}} \right) \cdot \left( {\nabla^{2} \sqrt n } \right)/\sqrt n } \right)$$

where *ħ* = *h*/2*π*, *h* is plank constant, *m*_*n*_ is an effective mass of the electron, *γ* (*γ* = 1.28) is a fitting parameter and *n* is electron density.

The surface traps were introduced as donor state (+ *σ*_D_) on the surface of AlGaN barrier layers Fig. 1a, and the simulation was carried out at the temperature of 300 K. The calibration done with the initial aluminum concentration of 28%.

## Simulation Results and Discussion

### Effect of Aluminum Percentage and Surface Traps on 2DEG Density

**Fig. 3:**
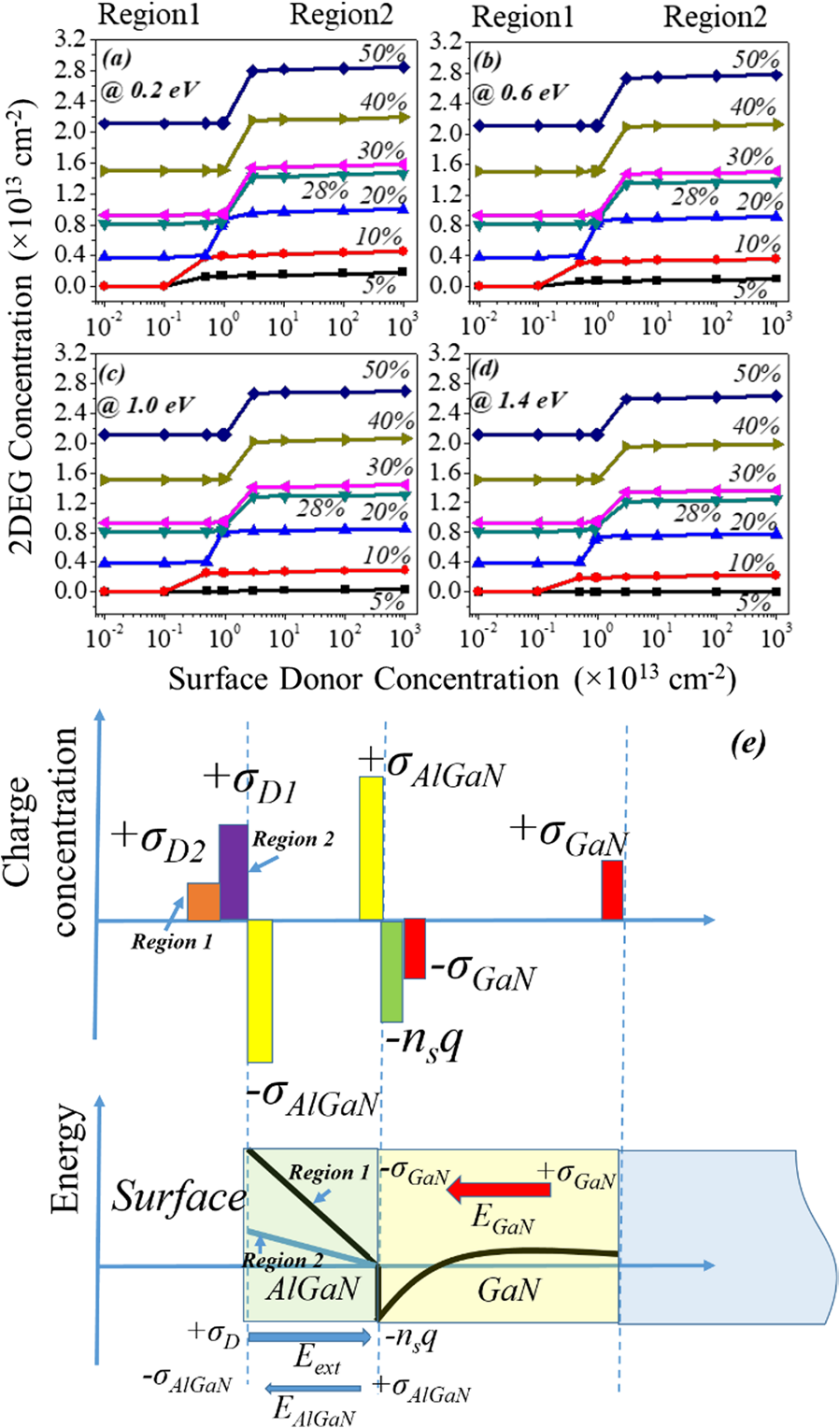
**a**–**d** Variation of electron sheet density in 2DEG w.r.t surface donor concentration and energy (from shallow 0.2 eV to deep 1.4 eV) for different aluminum percentage. **e** Different charge distribution and electric field orientation in the device

**Fig. 4:**
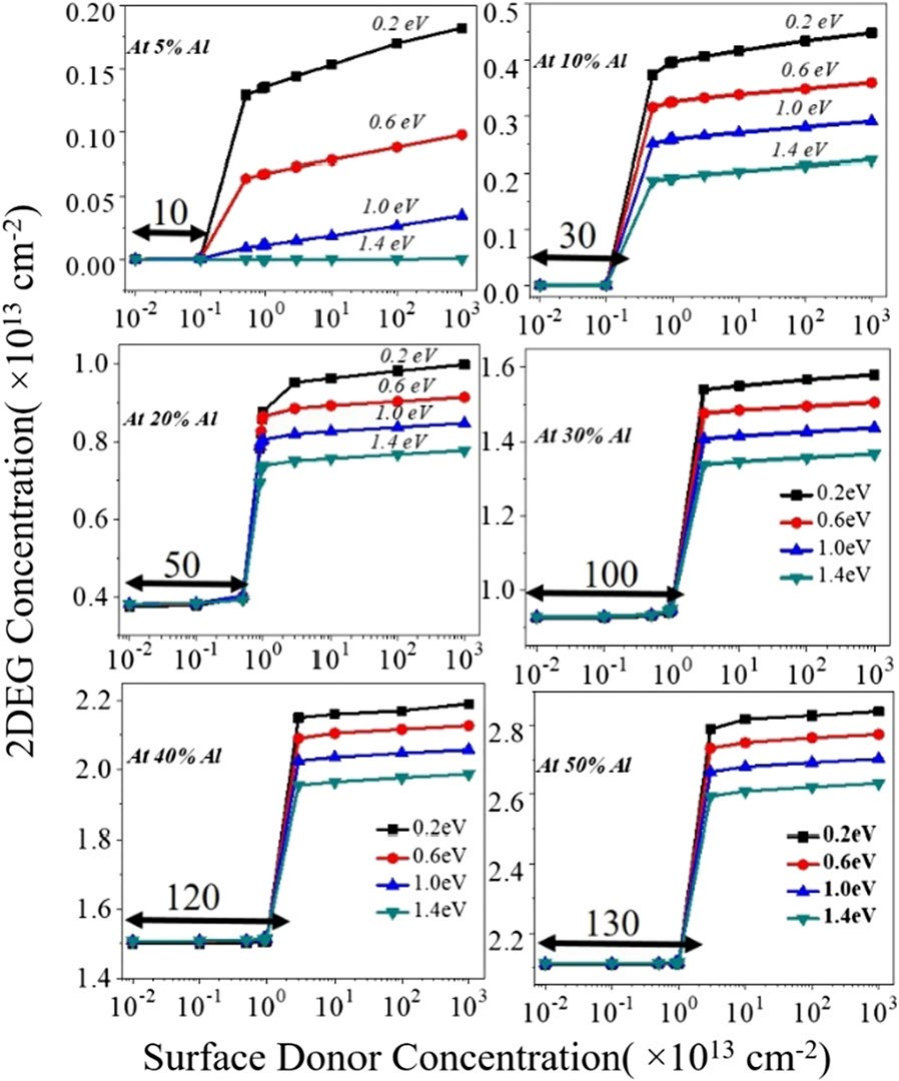
Variation of an individual aluminum percentage to donor surface trap from deep to shallow with respect to the conduction band

**Fig. 5:**
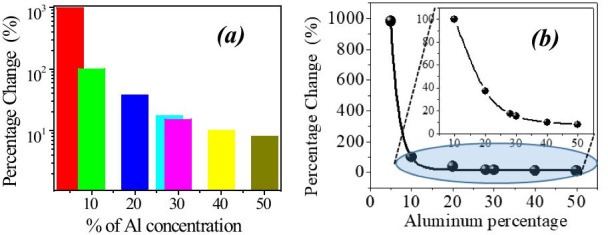
**a** Log scale percentage change in 2DEG electron density for Al concentration when the surface donor becomes shallow from deep level with respect to the conduction band. **b** Linear scale

**Fig. 6:**
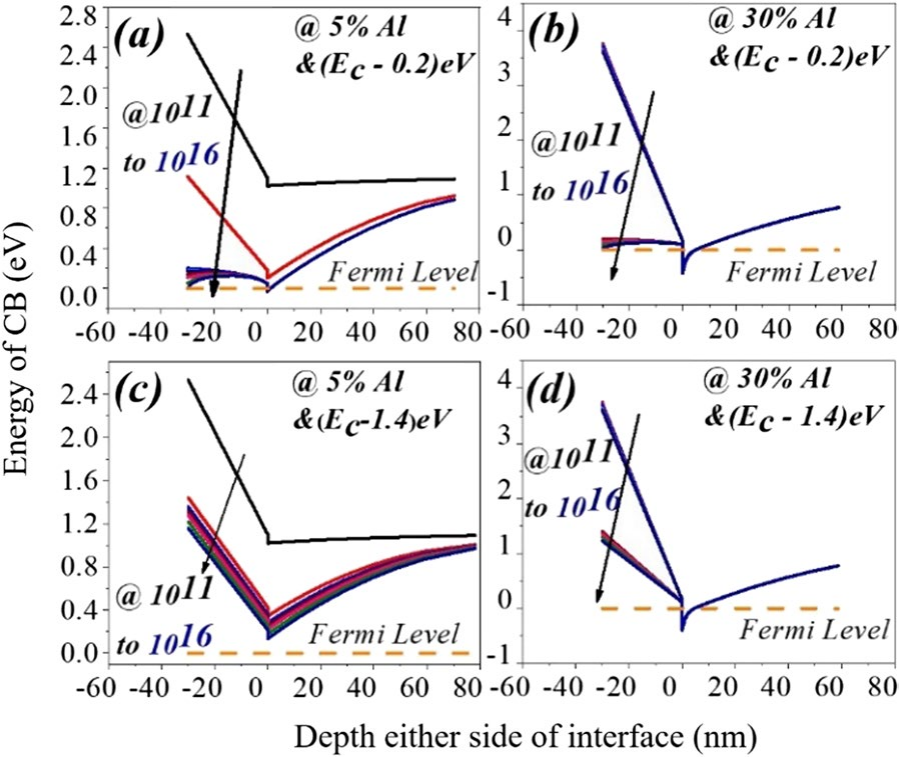
**a**, **c** Conduction band variation either side of the AlGaN–GaN interface for 5% aluminum and **b**, **d** for 30% aluminum. Deep level surface trap does not contribute electron and positive sheet charge to the potential well and surface, making conduction band slope higher. Even for deep donor surface state (1.4 eV) there is 2DEG potential well is forming for 30% aluminum. This is not true for 5% aluminum

The device was simulated under no applied bias conditions to investigate the 2DEG electron density. While we are concentrating on 2DEG electron density, for all energy of donor state, up to a certain value (relatively lower value) of donor trap concentration, there is no significant change in electron density (i.e., Region1). 2DEG electron density proportionally changes with surface donor concentration (between Region1 to Region2). After a certain threshold value of surface donor trap, again there is no change appearing in the electron density (i.e., Region2), see Fig. 3a–d. This mechanism can be explained as follows:

(i) The conduction band of the AlGaN barrier layer for region1 has a larger gap from the Fermi level at the surface. As donor trap concentration starts increasing from lower to a higher value, in the transition period (between Region1 to Region2), conduction band proportionally starts moving toward the Fermi level. Thus, 2DEG concentration starts increasing from a lower value to a higher value. In the transition period (between Region1 to Region2) conduction band proportionally starts moving toward the Fermi level and so the donor surface energy also moves toward Fermi level. For Region2, once donor concentration crosses the threshold value, bending of the conduction band starts in such a way that the energy of donor trap pins the Fermi level. Due to Fermi level pining all the donor states get ionized and contribute electrons to the 2DEG triangular quantum potential well. Once the energy of donor states get pinned to the Fermi level, no significant change reflects in the electron density. (ii) To find charge neutrality in the device, surface donor states are essential to counter the electrons in 2DEG. As surface donor states increases, an electric field starts to increase from surface to 2DEG quantum well. This electric field counters the built-in electric field produced by the polarization sheet charge (± *σ*_AlGaN_). When the external electric field starts exceeding the internal electric field (due to ± *σ*_AlGaN_), it brings down the conduction band at the surface and hence contributes more electron to the 2DEG potential well, see Fig. 3e. When aluminum percentage increases from 5 to 50%, polarization sheet charge density also proportionally increases, which leads to high internal electric field (due to polarization). To overcome this internal electric field, a higher concentration of surface donor traps is required. Hence, the transition region gets shifted (from 10 to 130 times with 10^11^ cm^−2^) for the higher value of donor trap concentration, where 2DEG electron density changes proportionally to the donor trap concentration, Fig. 3a–d. 2DEG concentration for each aluminum percentage with respect to the surface donor (concentrate + energy) is plotted in Fig. 4. In spite the pattern of 2DEG electron concentration is same for all percentages of aluminum when the donor trap goes from shallow (0.2 eV) to deep (1.4 eV) (Fig. 5), the change in 2DEG electron density from deep to shallow is still quite significant. In the case of 5% aluminum concentration, the donor trap goes from deep (1.4 eV) to shallow (0.2 eV), it does not contribute significant to the potential well. As polarization charge concentration (± *σ*) is of the order of 10^11^ cm^−2^ for 5% aluminum see Fig. 2a, the electric field due to these polarization charges are not enough to bring conduction band offset below the Fermi level, hence no 2DEG triangular potential well is formed at the GaN side of the AlGaN–GaN interface structure. It is also evident that even for the higher concentration of surface donor traps, the saturation of electron density does not occur as shown in Figs. 4a and 6. This is also true for 10% aluminum percentage as shown in Fig. 4b. For 20% and beyond, the polarization charge (± σ) concentration is greater than 10^12^ cm^−2^. So the internal electric field is large enough to pull conduction band offset below Fermi level and hence it forms the 2DEG triangular quantum potential well see Fig. 6b, c. So for 20% and above aluminum percentage, 2DEG electron density approaches to ~ 10^13^ cm^−2^ for shallow donor traps as shown in Fig. 4c. For aluminum concentration of 20%, 30% and beyond, the contribution of the electron in the triangular well is having density of 1 × 10^13^ to 3 × 10^13^ cm^−2^. Figure 5a,b depicts the percentage change of electron density in triangular well when donor trap energy changes from 1.4e to 0.2 eV. As we go from 5 to 50% aluminum percentage, the change in 2DEG concentration reduces significantly from 10.89 times to 1.08 times and gets saturated beyond 30%.

### Effect of Aluminum Percentage and Surface Trap on Surface Potential

**Fig. 7:**
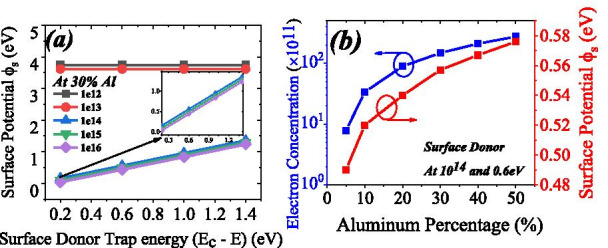
**a** Surface potential variation with respect to energy of surface donor trap. At lower concentration (less than 1e14) there is no significant change in surface potential. Surface donor concentration above 1e13, surface potential changing proportionally to the energy of surface donor. **b** Electron concentration in 2DEG changing from 7.79 × 10^11^ to 2.75 × 10^13^ for aluminum percentage changes to 50% from 5% (blue line). Surface potential changes approximately 0.1 eV from 5 to 50% of aluminum percentage

Some of the literatures have discussed the surface potential variation due to change in aluminum percentage [29]. But they have not incorporated the effect of surface donor traps on surface potential. Here we are reporting the variation of surface potential due to surface donor traps, in both the dimensions of energy and concentration, see Fig. 7a. In this study we have changed the surface donor concentration from 1 × 10^12^ to 1 × 10^16^ and surface donor energy from 0.2 to 1.4 eV. Surface potential has been calculated from Fig. 6b. Surface potential settles near 3.7 eV (for surface donor concentration 1 × 10^12^) and 3.6 eV (for surface donor concentration 1 × 10^13^). This surface potential does not depend on the energy of surface donor trap for its lower value. The surface potential linearly increases as surface donor goes deep (1.4 eV) from shallow (0.2 eV). As surface potential goes down, the 2DEG electron concentration will increase because surface potential linearly varies with surface donor trap energy. Aluminum percentage also have a great impact on the surface potential. Increasing the aluminum percentage from 5 to 50%, electron concentration increases from 7.79 × 10^11^ to 2.75 × 10^13^. Surface potential also increases from 0.49 to 0.576 eV when aluminum percentage changes from 5 to 50% see Fig. 7b. Thus, surface donor trap concentration and energy along with aluminum concentration have a great influence on the surface potential.

### Effect of Aluminum Percentage and Surface Trap on Conduction Band and 2DEG Potential Well

**Fig. 8:**
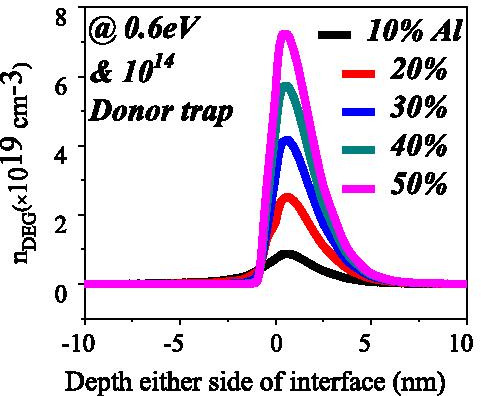
Electron density in triangular quantum potential well for different aluminum concentrations at fix donor concentration and energy

The ionized surface traps contribute electrons to the potential well and positive sheet charge at the AlGaN surface [6]. In case of 5% aluminum concentration, as traps go deep from shallow energy level, the amount of ionized surface traps become smaller and smaller. Thus, ionized surface traps contributed less electrons to triangular potential well and positive sheet charges at the surface. Less amount of positive sheet charges and electrons concentration in 2DEG does not contribute enough external electric field, and hence, the slope of the conduction band in the AlGaN layer becomes larger as shown in Fig. 6. This is also true for aluminum with 10% in AlGaN barrier layer. The free electrons from the surface donor states reside into 2DEG potential well, and they neutralize the positive sheet charge that appears at the surface of AlGaN. This electron sheet charge is calculated by [24]:

4 $$n_{{\text{s}}} (x) = \frac{ + \sigma (x)}{e} - \left( {\frac{{\varepsilon_{o} \varepsilon (x)}}{{de^{2} }}} \right)\left[ {e\phi_{{\text{b}}} (x) + E_{{\text{F}}} - \Delta E_{{\text{C}}} (x)} \right]$$

where *d* is the thickness of the Al_*x*_Ga_(1−*x*)_*N* barrier layer, *ϕ*_b_ is Schottky barrier, *E*_F_ is the Fermi level and Δ*E*_C_ is the conduction band offset at AlGaN–GaN interface. It is evident from Eq. (4) that electron sheet charge density is directly proportional to the conduction band offset and polarization charges which is a function of the aluminum percentage. As we increase the aluminum percentage from 10 to 50%, conduction band offset increases [25] and hence the electron density in 2DEG increases due to the increase in the number of energy levels see Fig. 8. The internal electric field of the device, when aluminum concentration is 20% and above, is such that the conduction band slope is high enough to construct the triangular potential well even for deep level (1.4 eV) surface trap energy and lower surface donor trap concentration as shown in Fig. 6b, d.

It is important to look at the notch of triangular quantum potential well-formed below the Fermi level ((*E*_F _− E) eV), where *E* is the energy below the Fermi level. Two important parameters in the triangular quantum potential well are the depth of potential well below the Fermi level and width of the potential well at the Fermi level. The confinement of the electrons in two-dimension regions is called the 2DEG quantum sheet. The density of states *N*(*E*) is one of the important features in the 2DEG quantum potential well. The density of states in two-dimensional quantum well is defined as [26]:

5 $$N(E) = \left( {{{m^{*} L_{{\text{X}}}^{2} E} \mathord{\left/ {\vphantom {{m^{*} L_{{\text{X}}}^{2} E} {\pi \hbar^{2} }}} \right. \kern-\nulldelimiterspace} {\pi \hbar^{2} }}} \right)$$

where m* is the effective mass of the electron and *L*_X_ is the width of well. This density of state in two-dimensional well looks like a step function.

**Fig. 9:**
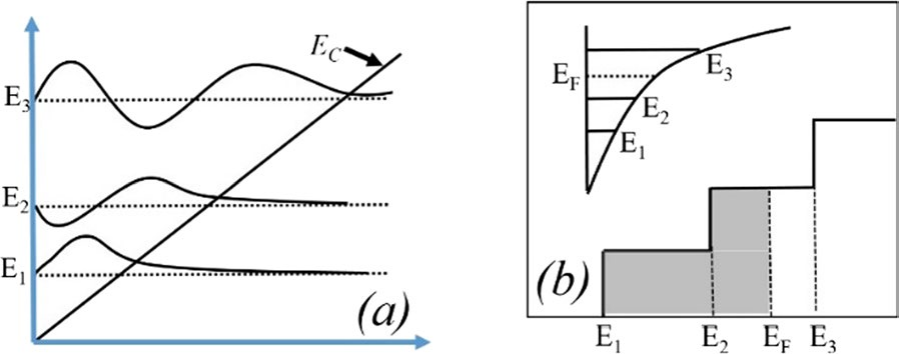
**a** A triangular potential well-depicting subbands energies with *Fang–Howard Airy* wavefunction. **b** Only lower energy subbands (*E*_1_ and *E*_2_, lower than Fermi level) are occupied [28]

**Fig. 10:**
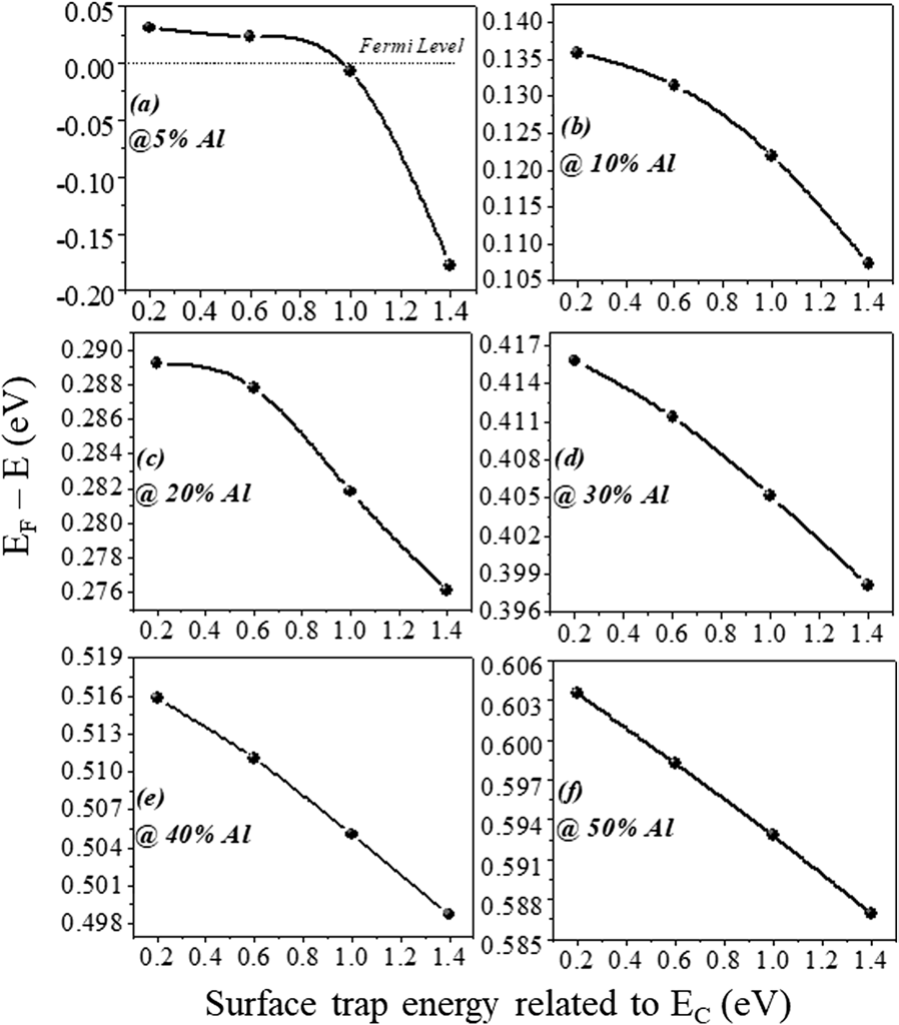
**a**–**f** (*E*_F_* − E*) variation with surface donor energy for all aluminum concentration

**Fig. 11:**
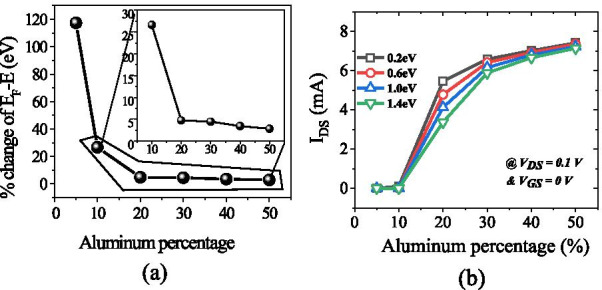
**a** The percentage change of energy *E* with aluminum concentration when surface donor energy changes deep to shallow. **b** Drain current and *V*_DS_ = 0.1 V and *V*_GS_ = 0 V at different surface donor traps energy. Up to 10% no significant current observed in the device

**Fig. 12:**
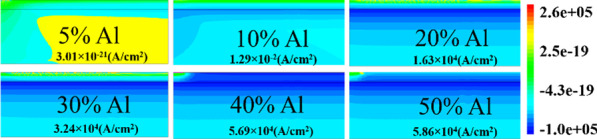
Contour plot of absolute total current density for 0.6 eV surface donor energy at *V*_DS_ = 0.1 V and *V*_GS_ = 0 V

**Fig.13:**
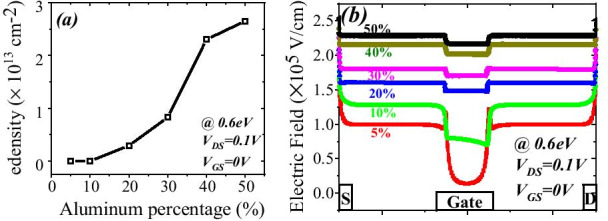
**a** Electron density variation with aluminum percentage and **b** electric field variation below gate and either side of gate for different aluminum percentage

The number of occupied states depends on the Fermi level. For example, if Fermi level is higher than *E*_1_ but less than *E*_2_; then only *E*_1_ subband is filled. If Fermi level is above *E*_2_, but less than *E*_3_, then two lower subband *E*_2_ and *E*_1_ are filled with electrons as shown in Fig. 9b. This signifies that if the energy at the interface goes deeper with the Fermi level, then only electrons will be expected in large number. In the AlGaN/GaN hetero-structure the energy spacing decreases ((*E*_2 _ − *E*_1_) > (*E*_3_ − *E*_2_)) for higher subbands [27]. As subband energy increases, the difference between them becomes negligible and looks continuous. The rigorously correct solution of the wave function contains the Poisson equation and the Schrodinger equation simultaneously. But the density-gradient model generates the approximate equal result with the Schrodinger equation. In quantum potential well, the energy level is quantified because this well forms up to a few nm lengths in the GaN side of the AlGaN–GaN interface. The deeper notch below the Fermi level will certainly have a higher number of quantified energy levels. The quantified energy level below the Fermi level is occupied. Hence, deeper the energy below the Fermi level, the electron concentration will be higher in 2DEG. From Fig. 9a, it is clear that the energy level below the Fermi level becomes higher when aluminum percentage increases because polarization charge increases and thus the internal electric field makes the notch to go deeper. As far as surface donor energy is concerned, it is evident from the previous discussion that when the surface traps go deeper (1.4 eV), the ionization of these surface donors reduces. Hence, the electric field is generated due to positive sheet charges at the surface and electrons contributed by these surface donors to 2DEG are not enough to overcome the internal electric field. Thus, the effect of polarization charge in terms of electric field reduces, which leads to less energy levels below the Fermi level. An exception is for 5% aluminum concentration, it is clear from Fig. 10a that value of *E*_F_ − *E* is negative as Fermi level is assumed at zero level, for the deep donor traps (> 0.9 eV to 1.4 eV), which signifies that the energy *E* is higher than the Fermi level (2DEG is not forming). For shallower surface donor traps (< 0.9 eV to 0.2 eV), the value of *E*_F_ − *E* is positive, which means that the value of the *E* is lower than the Fermi level. For rest of the aluminum concentration (10% to 50%), the value of *E*_F_ − *E* is positive which signifies that the value of *E* is lower than Fermi level and 2DEG notch is forming for all types of surface donor energy (from 0.2 eV to 1.4 eV). It is noted from Fig. 11a that the percentage change of energy *E* with aluminum gets saturated beyond 20% aluminum concentration, which is also in tune with Fig. 5. The depth of notch below Fermi level does not change significantly after 20% aluminum concentration when surface donor traps energy change from deep to shallow. Figure 11b also depicts that there is no significant current up to 10% aluminum mole fraction. Beyond 10% there is significant change in current when surface donor energy changes from *E*_C_ − 0.2 to *E*_C_ − 1.4 eV and saturated beyond 20% again. This result is also in tune with Figs. 11a and 5. The contour plot of absolute current density also shows that it saturates above 20% Al mole fraction and no significant current density till 10% of Al mole fraction Fig. 12. This also validates non-forming of 2DEG till 10% Al mole fraction. A significant amount of electron density observed above 20% of mole fraction Fig. 13a. Electric field distribution along the channel is plotted in Fig. 13b. Figure 13b shows that there is no notably improved electric field below gate till 10% of Al mole fraction and above 20% of Al mole fraction there is no much difference in electric field, which limits the current at higher Al percentage.

## Conclusion

In this paper, we comprehensively present the effect of surface donor traps along with aluminum percentage on electron density and quantum potential well. This manuscript demonstrated that the percentage change happens in 2DEG and notch below the Fermi level gets saturated above 20% of aluminum concentration when surface donor trap energy goes deep to shallow. The electron density in the two-dimensional quantum potential well is saturated approximately at 8%, whereas the energy below the Fermi level saturates somewhere around 2%. These two results are also in tune with each other, except 5% aluminum, having a condition for not forming two-dimensional well for relatively deep (> 0.9 eV) surface donors. Aluminum percentage above 10% forms two-dimensional quantum potential well even for deeper surface donor traps. The effect of surface donor traps on the surface potential also has been discussed in this work. The results of this paper may provide the impetus to the experimental result validation.

## Data Availability

All data are available on request.

## Abbreviations

GaN: Gallium nitride
HEMT: High-electron-mobility transistor
2DEG: Two-dimensional electron gas
DD: Drift and diffusion transport model
SRH: Shockley–Read–Hall recombination model
